# Comparing proximal convergence ratio in myopes

**DOI:** 10.22336/rjo.2021.71

**Published:** 2021

**Authors:** Undrakonda Vivekanand, Sarita Gonsalves Lobo, Navya Dharmappa, Alekya Kesireddy

**Affiliations:** * Department of Ophthalmology, Alluri Sitarama Raju Academy of Medical Sciences, Malkapuram, Eluru, Andhra Pradesh, India; ** Department of Ophthalmology, Father Muller’s Medical College, Mangalore, Karnataka, India

**Keywords:** AC/ A ratio, myopes, proximal convergence ratio

## Abstract

**Aim:** To determine if there is a difference at all in proximal convergence to accommodation ratio (PC/ A) between 2 myopic groups. Group A: -0.50D < SE < 3.00D, SE: spherical equivalent, D: Diopter) and Group B (-3.00D SE and above).

**Patients and methods:** In this prospective observational study, 24 consecutive patients with myopia between -0.50D < SE < 3.00D were included in group A and 27 consecutive patients with myopia > -3.00 D SE were included in group B. Patients with pathological myopia, strabismus or heterophoria anomalies were excluded. The PC/ A ratio for both the groups were calculated by measuring the difference between value of accommodative convergence to accommodation ratio (AC/ A) derived from heterophoria and gradient method using a prism bar method. Statistical analysis was performed by analyzing descriptive statistics and the independent sample t-test using SPSS ver. 22.0 software.

**Results:** The average calculated PC/ A ratio was 3.24∆/ D (SD = +0.68/ D) in group B (n=27) and 2.6∆/ D (SD = 0.86/ D) in group A (n=24) (p value <0.05).

**Conclusion:** An evident high AC/ A ratio in group B patients with myopia more than -3.00 D was the predominant cause for high PC/ A ratio in group B patients.

## Introduction

Convergence is one of the most important components of the near vision triad. It is a vergence or adduction movement that increases the visual angle to permit a single binocular vision during near viewing. There are several different types of convergences. These include proximal convergence, accommodative convergence, tonic convergence, and fusional convergence. Fusional convergence and tonic convergence are subtle and often incomparable to the actions of accommodative and proximal convergences. The ratio of accommodative convergence (AC) that occurs per diopter (D) of accommodative response (A) is referred to as AC/ A ratio [**1**].

Proximal convergence is stimulated by a sense of nearness. It appears to be initiated by psychological factors since it also occurs when a subject just believes that he or she is looking at the near object although he or she is not. It has been found that for about each diopter of change of fixation distance, an approximate change of 1.5 prism diopter occurs in proximal convergence.

Clinically, there are two methods of measuring AC/ A ratio: calculated (heterophoria method) AC/ C ratio and gradient AC/ A ratio. The calculated AC/ A ratio is derived from the measurement of near and phorias at distance, based on interpupillary distance (PD) and the alterations of convergence capacity as the accommodation are stimulated by clear near the target. The gradient AC/ A ratio is based on the convergence response to the addition or reduction of spherical lenses when the eyes are near sightedly stimulated. The difference between the two is used to quantify the amount of proximal convergence [**1**-**3**]. 

Earlier studies have documented that a greater amount of myopia has a higher AC/ A ratio than that of lower myopia. This study aims at measuring PC/ A ratio changes seen in patients with myopia greater than -3.00 D.

## Patients and methods

In this prospective observational study, 24 consecutive patients with myopia between -0.50D < SE < 3.00D (SE: Spherical equivalent, D: Diopters) were included in group A and 27 consecutive patients with myopia > -3.00 D SE were included in group B. All patients with age <40 years were recruited in this study. The study was conducted as per the guidelines of the Declaration of Helsinki and clearance from Alluri Sitarama Raju Ethical Committee was obtained before initiation (IEC/ ASR/ APPROVAL/ 05/ 2019). The study was registered in ctri.nic.in -CTRI/ 2019/ 05/ 019256 (Registered on 21/ 05/ 2019). Following this,informed consent was obtained in the local language from all the patients included in this study. Patients with pathological myopia, strabismus or heterophoria anomalies were excluded. Documentation of the variables that were included were age, gender, measurement of Snellens’ fraction visual acuity (later converted to log MAR) and accommodative convergence ratio (AC/ A) by both heterophoria (calculated) and gradient method using prism bar method. 

*Measurement of calculated AC/ A ratio by heterophoria method*

After assessing best-corrected visual acuity (BCVA), The AC/ A ratio was calculated as AC/ A = IPD (cm) + N (m) (D’-D).

Accommodative convergence (AC) that occurs per diopter (D) of accommodative response (A) was referred to as AC/ A ratio (IPD = Interpupillary distance in centimeters, N = Near fixation distance in meters (m), D’ = Near phoria (eso is plus and exo is minus), D = Far phoria (eso is plus and exo is minus). The recorded unit was ∆/ D).

*Measurement of gradient AC/ A ratio/*

After performing the above procedure, phoria was measured a second time using a -1.00/ +1.00 lens. The change in phoria with the additional minus or plus was the AC/ A ratio. This AC/ A ratio represented as gradient AC/ A, which was (∆0–∆l)/ D, where ∆0 was the amount of near lateral phoria, ∆l the amount of near lateral phoria after simultaneous addition of spherical lenses to both eyes, and D the added power of the spherical lens, with plus spherical lens being positive and with minus spherical lens being negative. The recorded unit was ∆/ D.

The difference between the two was used to quantify the amount of proximal convergence (PC). For statistical analysis variables such as age, the spherical equivalent of both group A and group B myopes, PC/ A ratio in both the groups were used. Statistical analysis was performed using descriptive analysis and the independent sample t-test. A p-value of <0.05 was considered statistically significant. SPSS ver. 22.0 (SPSS Inc., Chicago, IL, USA) was used.

## Results

**Tabel 1:** Comparing descriptive elements and PC/ A ratio in both groups

	Group A	Group B	P-value
No. of subjects	24	27	
Age (in years) + Std	22.4 + 7.9	22.1 + 5.5	
Sex (Male: Female)	13:11	15:12	1.00
Mean spherical equivalent (Std)	1.56(0.73)	6.09(1.2)	
Average PC/ A ratio in ∆ (std)	2.6(0.86)	3.24(0.68)	0.00
Std = Standard deviation, AC/ A = Accommodative convergence/ Accommodation, PC/ A ratio = Proximal convergence/ Accommodation			

The mean age of patients in group A and group B was 22.4 + 7.9 and 22.1 + 5.5 years respectively. The male to female ratio in group A and group B was 13:11 and 15:12 respectively. The results of calculated PC/ A ratios in the different types of myopia groups are listed in **Table 1**. The average calculated PC/ A ratio was 3.24∆/ D (SD = +0.68/ D) in group B (n=27), and 2.6∆/ D (SD = +0.86/ D) in group A patients (n=24). 

## Discussion

In previous reports, higher AC/ A ratio have been found among higher myopes. Furthermore, it has also been observed that higher accommodative lag and abnormal eye movements are risk factors of myopia [**4**,**5**]. An appreciable difference between calculated heterophoria and gradient AC/ A ratios has been suggested due to additive effect of induced proximal convergence and accommodative lag [**6**-**8**]. Myopes having elevated AC/ A ratios have been highlighted in many previous reports [**7**,**8**]. Effect of a near-vision task on the response AC/ A of a myopic population was demonstrated in some studies, where in excess near work could be a factor for myopia progression and higher AC/ A ratio [**9**,**10**]. Moreover, a significantly higher AC/ A ratio has been documented before and after the onset of myopia in children [**11**]. 

In our study, we found a significant difference in PC/ A ratio between group B as compared to group A patients, which was consistent with previous studies. The normal AC/ A ratio was between 3 and 6, regardless of the method of testing that was used. Nevertheless, values above 6 usually indicated an excess of convergence per unit of accommodation, whereas values below 3 suggested convergence insufficiency. The difference of PC/ A ratio between both groups could be attributed to differential modulations between stimuli and responses among the groups [**12**]. However, the inability to find an agreement between prism bar cover test and standard von Graefe method using a phoropter in all types of myopes could be a major limitation of this study [**13**].

## Conclusion

Hence, in patients having myopia more than -3.00 D, the AC/ A ratio needs to be interpreted with caution. Both AC/ A and PC/ A ratio are required before interpreting the results.

**Conflict of Interest statement**

The authors state no conflict of interest.

**Informed Consent and Human and Animal Rights statement**

Informed consent has been obtained from all individuals included in this study.

**Authorization for the use of human subjects**

Ethical approval: The research related to human use complies with all the relevant national regulations, institutional policies, is in accordance with the tenets of the Helsinki Declaration, and has been approved by Alluri Sitarama Raju Ethical Committee before initiation (IEC/ ASR/ APPROVAL/ 05/ 2019).

**Acknowledgements**

All the authors have substantial contributions to conception and design, analysis, and interpretation of data, drafting the article, revising it critically for important intellectual content and final approval of the version to be published.

**Sources of Funding**

None.

**Disclosures**

None.
